# Reference values for fetal Doppler-based cardiocirculatory indices in monochorionic-diamniotic twin pregnancy

**DOI:** 10.1186/s12884-021-04255-w

**Published:** 2021-11-30

**Authors:** Thananan Chongsomboonsuk, Nisarat Phithakwatchara, Katika Nawapun, Sommai Viboonchart, Suparat Jaingam, Tuangsit Wataganara

**Affiliations:** Siriraj Fetal Therapy Center (SiFTC), Division of Maternal-Fetal Medicine, Department of Obstetrics and Gynecology, Faculty of Medicine Siriraj Hospital, Mahidol University, 2 Prannok Road, Siriraj, Bangkoknoi, Bangkok, 10700 Thailand

**Keywords:** Cardiac function, Doppler indices, Gestational age, Monochorionic diamniotic twins, Reference values

## Abstract

**Background:**

Placental anastomoses in monochorionic diamniotic (MCDA) twin pregnancy have a major impact on fetal circulation. This study was designed to define reference ranges of cardiac and vascular Doppler indices in MCDA twin pregnancies.

**Methods:**

This cross-sectional study included 442 uncomplicated MCDA twin fetuses undergoing Doppler ultrasonography at 18–35 weeks of gestation. Left and right myocardial performance index (LV-MPI, RV-MPI), E/A ratio of atrioventricular valves, pulsatility indices of umbilical artery, middle cerebral artery (MCA), and ductus venosus (DV), cerebroplacental ratio, peak systolic velocity of MCA, S/a ratio of DV, and early diastolic filling time of ductus venosus (DV-E) were evaluated under standardized settings. The equation models between Doppler indices and gestational age (GA) were fitted. After adjustment for GA, the correlations between MPI and fetal heart rate (FHR), and between MPI and DV indices were analyzed.

**Results:**

Estimated centiles of Doppler indices were derived as a function of GA, being distinct in values from those of singletons. There was no correlation between GA-adjusted MPI and FHR. DV-E was inversely related to LV-MPI.

**Conclusions:**

MCDA twins showed significant changes in some Doppler indices throughout gestation with quantitative differences from singletons, emphasizing the importance of MC twin-specific reference values for clinical application. Further adjustment of MPI for FHR was unnecessary. DV-E is a vascular index indirectly representing fetal diastolic function.

**Supplementary Information:**

The online version contains supplementary material available at 10.1186/s12884-021-04255-w.

## Background

Monochorionic diamniotic (MCDA) twin pregnancies have a substantial proportion of cases developing severe complications including twin-to-twin transfusion syndrome (TTTS), selective fetal growth restriction (sFGR), twin anemia polycythemia sequence (TAPS), and twin reversed arterial perfusion sequence (TRAPS) [1–4]. These conditions alter fetal hemodynamics and occasionally cardiac function. Doppler ultrasonography is generally used for fetal surveillance to measure various cardiac and vascular indices. Previous studies have introduced Doppler indices for the diagnosis and management of complications associated with MCDA twin pregnancy, especially TTTS and sFGR [4–10]. The recipient in TTTS usually develops cardiac diastolic dysfunction, while the donor has increased vascular resistance due to renin-angiotensin-aldosterone system upregulation. In cases treated with fetoscopic laser surgery, the recipient’s cardiac function returns to normal and the donor may have transient cardiac impairment postoperatively [11, 12]. The fetal myocardial performance index (MPI) and E/A ratio of flow across the atrioventricular valve are common parameters for cardiac function assessment, whereas umbilical artery (UA), middle cerebral artery (MCA), and ductus venosus (DV) waveforms are customarily evaluated in MCDA twin pregnancies. Typically, the reference values of Doppler indices are fundamental in daily practice [11]. Placental anastomoses in MC twin pregnancy have a major impact on fetal circulation, deviating from singleton pregnancy [3, 4]. Thus the adoption of normative values of Doppler indices specific to MCDA twin pregnancy is a prerequisite.

Only a few studies in literature have examined normal Doppler indices in MCDA twin pregnancies with distinct study designs, ultrasound settings, and statistical analyses [11, 13–15]. Thus, we performed a study on a prospective cohort of MCDA twin fetuses under a well-suited ultrasound setting to construct normative values of cardiac and vascular Doppler indices pragmatically used for surveillance. As the effect of fetal heart rate (FHR) on MPI is still uncertain, the relationship between FHR and MPI was also determined [16]. Furthermore, we explored whether any DV indices correlated with MPI to find the vascular index indirectly representing cardiac diastolic function.

## Methods

### Study design and population

This cross-sectional study was conducted at the Division of Maternal-Fetal Medicine, Department of Obstetrics and Gynecology, Faculty of Medicine Siriraj Hospital from October 2019 to March 2021 with Siriraj Institutional Review Board approval. The study was supported by a grant from the Faculty of Medicine Siriraj Hospital, which had no role in the study design, data collection, statistical analysis and interpretation, manuscript preparation, or publication decision. A sample size of 50 fetuses per 2-week gestational period was calculated using a significance of 5%, standard deviation of 30%, and margin of error in estimating the mean of 10% [11]. A total of 450 MCDA twins from 225 women at 18–35 weeks of gestation were consecutively recruited with written informed consent. All patients were aged 18 years or older, without chronic diseases. Gestational age was estimated by either a reliable menstrual history and/or by ultrasound examination of fetal crown-rump length before 14 weeks of gestation. The chorionicity was confirmed using sonographic biometry during the first trimester. Those with fetal abnormality, complicated MCDA twin pregnancy (TTTS, sFGR, TAPS, or TRAPS), fetal demise, or maternal complications during pregnancy were excluded from the data analysis.

### Fetal Doppler waveforms acquisition

**Fig. 1:**
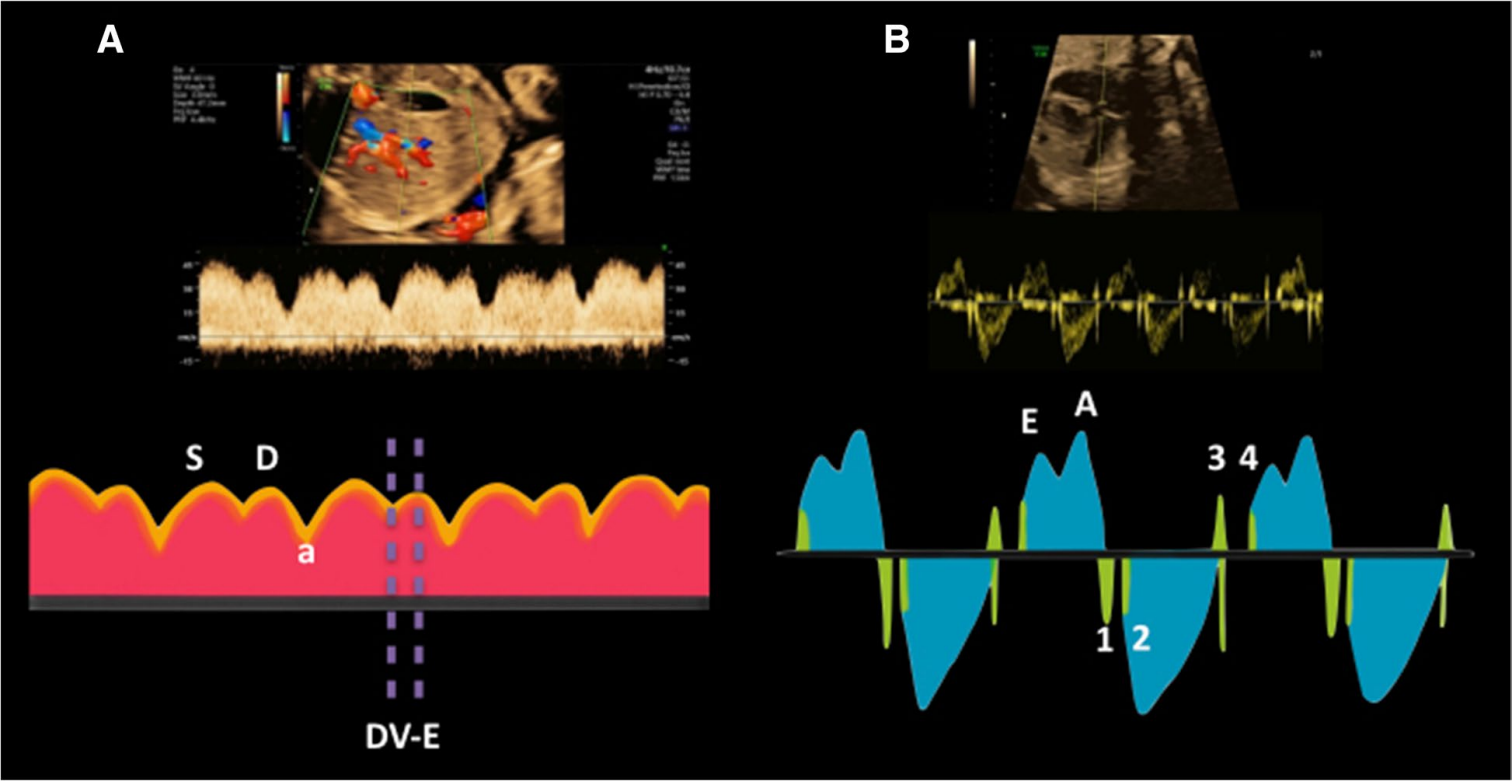
**A** Early diastolic filling time measured in ductus venosus Doppler waveforms (DV-E) (S = systolic wave, D = diastolic wave, and a = atrial wave); **B** Left myocardial performance index (LV-MPI) Doppler waveforms, 1 = mitral valve closure click, 2 = aortic valve opening click, 3 = aortic valve closure click, and 4 = mitral valve opening click (E = peak velocity in early diastole, A = peak velocity in late diastole by atrial contraction)

All Doppler waveforms were obtained at a fetal heart rate of 120–160 beats/min in the absence of fetal movements, using a Voluson E10 ultrasound machine (GE Medical Systems, Tiefenbach, Austria) with a 2–8 MHz abdominal transducer (RAB6-D H48681MG). According to the ISUOG practice guidelines, Doppler indices of UA, MCA, and DV were sampled just distal to the abdominal insertion of the umbilical cord [17], at the proximal one-third of the MCA [18], and at the origin of the DV in the mid-sagittal or transverse abdominal plane [17], respectively. The ultrasound beam was parallel to the direction of blood flow with an angle close to 0^o^. The machine automatically computed pulsatility indices of UA (UA-PI) and MCA (MCA-PI), pulsatility index for veins (PIV) and systolic/atrial wave (S/a) ratio of DV (DV-PIV and DV-S/a). The cerebroplacental ratio (CPR), defined as the ratio of MCA-PI to UA-PI, was then calculated [19]. The peak systolic velocity was measured at the top of MCA waveform (MCA-PSV). The E-wave duration of DV (DV-E) was estimated as the time between ventricular end-systole (v-descent) and ventricular diastole (D-wave) (Fig. 1A) [20].

To evaluate the E/A ratios of the left and right ventricles (LV-E/A and RV-E/A), the pulsed Doppler sample volume was placed at the tips of the mitral and tricuspid leaflets in the apical four-chamber view. For left MPI (LV-MPI), the target position of 3–4 mm Doppler sample volume was on the lateral wall of the ascending aorta, at a level below the aortic valve (AV) and just above the mitral valve (MV) [21]. The angle of insonation was kept at less than 30^o^. The Doppler gain was decreased to − 10 dB to make the valve clicks clearer on the Doppler waveform. The spectral Doppler was set at a sweep speed of 5 (velocity of 15 cm/s) and WMF of 210 Hz [22]. The duration of the isovolumetric contraction time (ICT), isovolumetric relaxation time (IRT), and ejection time (ET) were measured in relation to MV and AV clicks. Originally, the valve closure click appeared on the opposite side of the flow waveform, whereas the valve opening click appeared on the same side of the flow waveform. ICT and IRT were estimated from the MV closure click to the AV opening click and the AV closure click to the MV opening click, respectively. In the meantime, ET was estimated from the AV opening click to the AV closure click. In this study, two different caliper positions were applied for ICT, IRT, and ET measurements: (1) from the beginning of valve click to the beginning of the subsequent one which was the original method (oICT, oIRT, oET) [21], and (2) from the peak of valve click to the peak of the subsequent one (pICT, pIRT, pET) (Fig. 1B) [16]. LV-MPI was calculated as the proportion of total isovolumetric contraction and relaxation time (ICT + IRT) to ET.

For right MPI (RV-MPI), right ventricular inflow and outflow were separately assessed in two different cardiac cycles due to anatomical separation of the tricuspid and pulmonary valves (TV and PV). The machine setting was similar to that of the LV-MPI assessment. In the apical four-chamber view, the Doppler sample volume was positioned at the tips of the TV leaflets to measure the time from the peak of the TV closure click to the peak of the TV opening click (T interval, TI). In the short-axis view, the Doppler sample volume was positioned at the PV to measure the time from the peak of the PV opening click to the peak of the PV closure click (pulmonary flow, PF). The fetal heart rate disparity between the TI and PF assessments was limited to less than 5 beats/min. RV-MPI was calculated as the proportion of the difference between TI and PF (TI –PF) to PF.

Each of the Doppler indices was determined three times and the average value was used for data analysis. The mechanical and thermal indices were less than 1. All scans were only utilized for the purpose of the research study. The spectral Doppler study of each fetus was performed by one of the three operators. All three operators had more than 65 MPI evaluations before participating in this study [23]. The inter- and intra-observer reliabilities of the MCA-PSV, DV-E, MV-E/A, TV-E/A, ICT, IRT, ET, TI, PF, LV-MPI, and RV-MPI were also assessed. For the intra-observer reliability, these indices of 10 fetuses were performed three times by the same operator with a 5-min interval, and a part of the ultrasound screen was covered with paper to blind the results to the operator. In another 10 fetuses, the stored images of these indices were used for offline measurements by all three operators to determine inter-observer reliability.

Of all cases recruited to the study, the information of the perinatal outcomes, noted by obstetricians and neonatologists in the medical records, was obtained and recorded in a computer database for analysis.

### Statistical analysis

Maternal characteristics and perinatal outcomes were described as number (percentage) for categorical data and mean ± standard deviation or median and interquartile range (IQR), depending on the normality of data. Regression analysis was used to analyze the relationship between each Doppler index and gestational age. A Gaussian distribution of the data was determined using the D’Agostino-Pearson omnibus test. Logarithmic transformation was applied to non-Gaussian distributed indices. The best-fitting model for each index was considered. The 5th, 50th, and 95th percentiles of each gestational week were derived from the regression equation. The reference values were compared to those of singleton and MCDA twin fetuses from previous studies. The reproducibility of each index was assessed by calculating the intraclass correlation coefficients (ICCs) of inter- and intra-observer reliabilities. Between the two methods of caliper placement for LV-MPI measurement, the differences, correlation, and agreement were tested using the Wilcoxon signed-rank test, Spearman’s rank-correlation coefficient, and Bland-Altman analysis, respectively. The raw data of the Doppler indices were transformed into z-scores adjusted for gestational age. The correlations between variables were then explored using Spearman’s rank-correlation coefficients. A two-tailed significance level of 5% was used for all tests. All analyses were performed using SPSS (IBM SPSS Statistics for Windows version 18, Microsoft Corporation; Chicago, IL, USA) and GraphPad Prism (GraphPad software for Windows version 7.00, San Diego, CA, USA).

## Results

A total of 450 fetuses were enrolled in the study, and the population analyzed was restricted to 442 fetuses (Table S1). Of the eight fetuses excluded, two pairs of twin fetuses developed sFGR, one twin pair had sFGR together with maternal preeclampsia, and another pair had maternal preeclampsia. Maternal characteristics and perinatal outcomes are shown in Table 1. All patients were Asian and none was reported as a current smoker.

**Table 1:** Maternal characteristics and delivery outcomes of the study population

	Median (IQR) or n (%)
**Maternal age (years)**	29 (27–34)
**Maternal BMI (Kg/m**^**2**^**)**	22.2 (19.9–23.2)
**Parity**	
**nulliparity**	119 (53.8)
**multiparity**	102 (46.2)
**Method of conception**	
**natural**	192 (86.9)
**IVF**	29 (13.1)
**Gestational age at delivery (weeks)**	36 (34–37)
**Birth weight (grams)**	2240 (2095–2390)

*BMI* body mass index, *n* number, *IQR* interquartile range, *IVF* in vitro fertilization

### The relationship between Doppler-based cardiocirculatory indices and gestational age

**Fig. 2:**
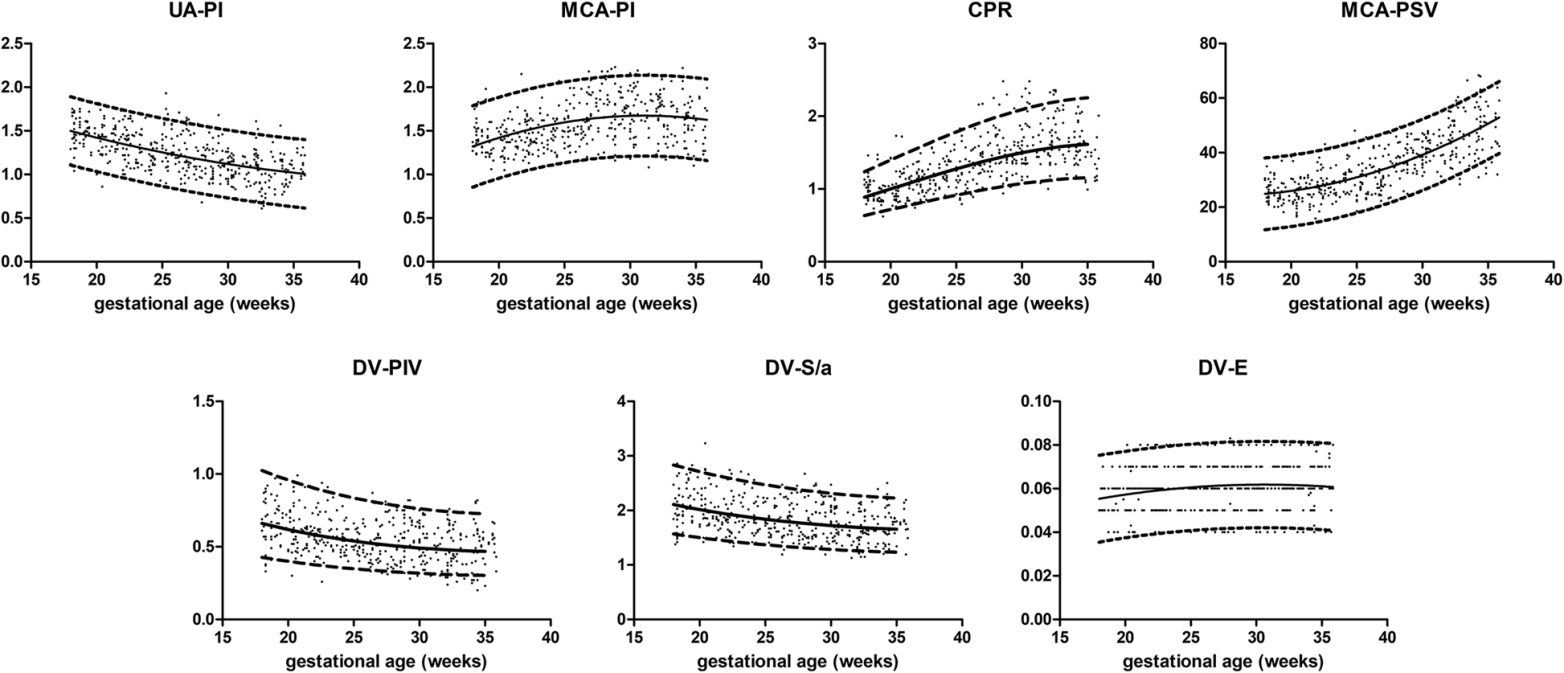
Scatter plots with the 5th, 50th, and 95th percentiles of vascular Doppler indices as a function of GA. The superimposed dots on the plot represent the raw values. *(UA-PI, pulsatility index of umbilical artery; MCA-PI, pulsatility index of middle cerebral artery; CPR, cerebroplacental ratio; MCA-PSV, peak systolic velocity of middle cerebral artery (cm/sec); DV-PIV, pulsatility index for vein of ductus venosus; DV-S/a, systolic/atrial wave ratio of ductus venosus; DV-E, early diastolic filling time of ductus venosus (sec))*

**Fig. 3:**
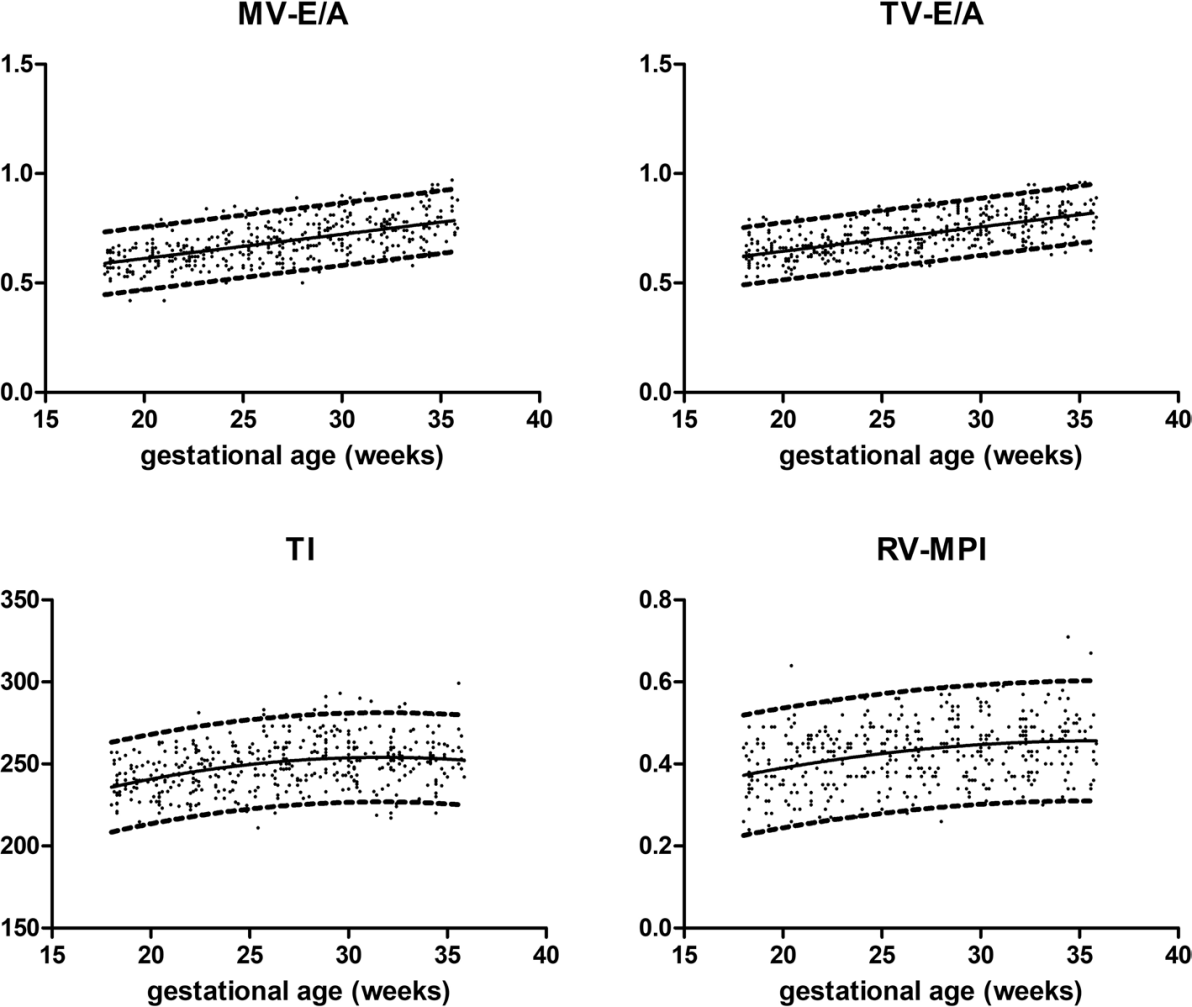
Scatter plots with the 5th, 50th, and 95th percentiles of the E/A ratio and right myocardial performance index as a function of GA. The superimposed dots on the plot represent the raw values. *(MV-E/A, E/A ratio of flow across mitral valve; TV-E/A, E/A ratio of flow across tricuspid valve; TI, time from tricuspid valve closure to tricuspid valve opening (sec); RV-MPI, myocardial performance index of right ventricle)*

**Fig. 4:**
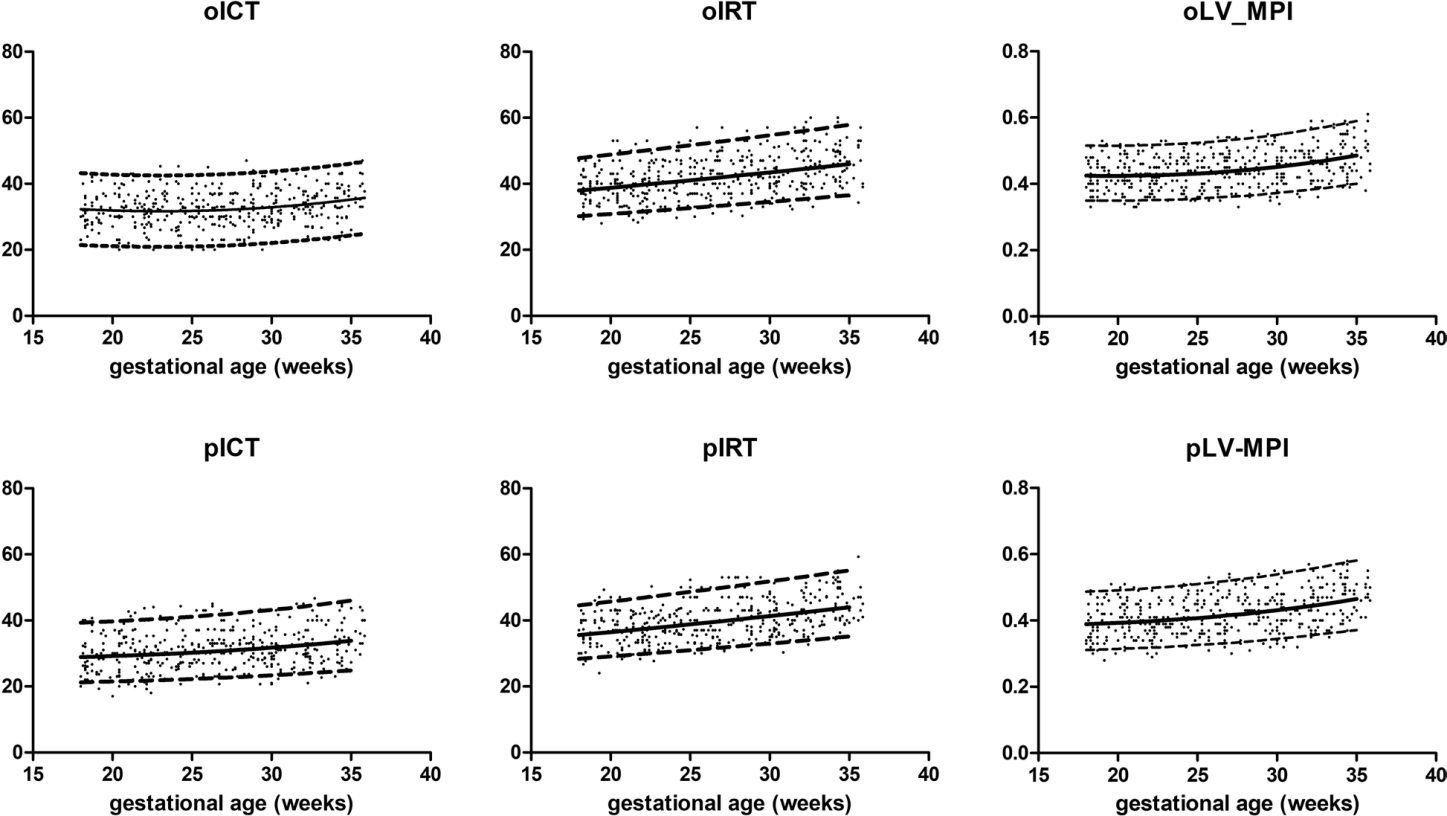
Scatter plots with the 5th, 50th, and 95th percentiles of the left myocardial performance index as a function of GA. The superimposed dots on the plot represent the raw values. *(oICT, isovolumetric contraction time measured by placing the caliper at the beginning of valve clicks (sec); pICT, isovolumetric contraction time measured by placing the caliper at the peak of valve clicks (sec); oIRT, isovolumetric relaxation time measured by placing the caliper at the beginning of valve clicks (sec); pIRT, isovolumetric relaxation time measured by placing the caliper at the peak of valve clicks (sec); oLV-MPI, myocardial performance index of left ventricle measured by placing the caliper at the beginning of valve clicks; pLV-MPI, myocardial performance index of left ventricle measured by placing the caliper at the peak of valve clicks)*

All Doppler indices were obtained from the entire cohort. The regression models of Doppler indices are presented in Table 2 and the reference ranges are shown in Figs. 2, 3 and 4 and Table S2, S3, S4 and S5. UA-PI decreased with advancing gestation, whereas MCA-PI increased until 32 weeks, contributing to the significantly increased CPR (*P* < 0.001 for all). MCA-PSV also increased during these gestational weeks with a dramatic increase after 25 weeks. DV-PIV and DV-S/a decreased as the gestational age increased, with a slower rate after 32 weeks. In the meantime, DV-E time gradually increased from 18 to 28 weeks and became stable thereafter.

**Table 2:** The regression equations of Doppler indices as a function of gestational age in weeks

Doppler index	Transformation	Constant	SD	r^**2**^	***P***-value	95% CI around regression slope coefficients
**UA-PI**	–	2.441–0.0648GA + 0.0007GA^2^	0.1981	0.349	< 0.001	− 0.1063 – − 0.0233 for GA − 0.0001 - 0.0015 for GA^2^
**MCA-PI**	–	− 0.325 + 0.1286GA - 0.0021GA^2^	0.2358	0.169	< 0.001	0.0792–0.178 for GA − 0.003 - -0.0011 for GA^2^
**CPR**	log_10_	− 0.7936 + 0.0544GA – 0.0007GA^2^	0.0882	0.45	< 0.001	0.036–0.073 for GA − 0.0011 - -0.0004 for GA^2^
**MCA-PSV**	–	38.4–1.917GA + 0.0647GA^2^	6.651	0.595	< 0.001	−3.311 – − 0.5235 for GA 0.0387–0.0907 for GA^2^
**DV-PIV**	log_10_	0.2189–0.029GA + 0.0004GA^2^	0.1156	0.135	< 0.001	− 0.0533 – 0.0048 for GA − 0.0001 - 0.0008 for GA^2^
**DV-S/a**	log_10_	0.5817–0.0185GA + 0.0002GA^2^	0.078	0.143	< 0.001	− 0.0349 – − 0.0022 for GA − 0.0001 - 0.0005 for GA^2^
**DV-E**	–	0.0235 + 0.0025GA - 0.00004GA^2^	0.0101	0.035	< 0.001	0.0004–0.0046 for GA − 0.00008 - -0.000001 for GA^2^
**MV-E/A**	–	0.391 + 0.011GA	0.0728	0.380	< 0.001	0.0097–0.0124 for GA
**TV-E/A**	–	0.422 + 0.011GA	0.0666	0.426	< 0.001	0.0099–0.0124 for GA
**oICT**	–	44.82–1.138GA + 0.0247GA^2^	5.51	0.038	< 0.001	− 2.292 – 0.0166 for GA 0.0031–0.0463 for GA^2^
**oIRT**	log_10_	1.491 + 0.0049GA	0.0607	0.147	< 0.001	0.0038–0.006 for GA
**oLV-MPI**	log_10_	−0.2757 - 0.0098GA + 0.0003GA^2^	0.0512	0.122	< 0.001	− 0.0206 – 0.0009 for GA 0.00005–0.00045 for GA^2^
**pICT**	log_10_	1.468–0.0026GA + 0.0001GA^2^	0.0812	0.063	< 0.001	− 0.0196 – 0.0144 for GA − 0.0002 - 0.0004 for GA^2^
**pIRT**	log_10_	1.453 + 0.0054GA	0.0596	0.181	< 0.001	0.0044–0.0065 for GA
**pLV-MPI**	log_10_	− 0.3855 - 0.0044GA + 0.0002GA^2^	0.0592	0.141	< 0.001	−0.0168 – 0.0008 for GA − 0.00006 - 0.0004 for GA^2^
**TI**	–	156.4 + 6.161GA - 0.0972GA^2^	13.84	0.139	< 0.001	3.26–9.062 for GA − 0.1514 - -0.043 for GA^2^
**RV-MPI**	–	0.1191 + 0.0188GA - 0.0003GA^2^	0.0742	0.107	< 0.001	0.0033–0.0343 for GA − 0.0006 - 0.00003 for GA^2^

*CPR, cerebroplacental ratio; CI, confidence interval; DV-E, early diastolic filling time of ductus venosus (sec); DV-PIV, pulsatility index for vein of ductus venosus; DV-S/a, systolic/atrial wave ratio of ductus venosus; GA, gestational age (weeks); MCA-PI, pulsatility index of middle cerebral artery; MCA-PSV, peak systolic velocity of middle cerebral artery (cm/sec); MV-E/A, E/A ratio of flow across mitral valve; oICT, isovolumetric contraction time measured by placing the caliper at the beginning of valve clicks (sec); pICT, isovolumetric contraction time measured by placing the caliper at the peak of valve clicks (sec); oIRT, isovolumetric relaxation time measured by placing the caliper at the beginning of valve clicks (sec); pIRT, isovolumetric relaxation time measured by placing the caliper at the peak of valve clicks (sec); oLV-MPI, myocardial performance index of left ventricle measured by placing the caliper at the beginning of valve clicks; pLV-MPI, myocardial performance index of left ventricle measured by placing the caliper at the peak of valve clicks; PF,; time from pulmonary valve opening to pulmonary valve closure (sec); RV-MPI, myocardial performance index of right ventricle; TI, time from tricuspid valve closure to tricuspid valve opening (sec); TV-E/A, E/A ratio of flow across tricuspid valve; r*^*2*^*, coefficient of determination; SD, standard deviation; UA-PI, pulsatility index of umbilical artery*

The MV-E/A and TV-E/A ratios steadily increased throughout the gestational period. oICT, pICT, oIRT, and pIRT were positively correlated with gestational age. Dissimilarly, oET and pET remained constant irrespective of gestational age (168.5 + 8.64 ms and 169.9 + 8.75 ms). In addition, TI and RV-MPI significantly increased with stable PF (174.7 + 10 ms) throughout gestation.

The median values of each Doppler index were plotted against gestational age with comparison to those from previous studies in singleton and MCDA twin fetuses, as illustrated in Fig. S1, S2 and S3. The inter- and intra-observer reliabilities of each index are shown in Table 3.

**Table 3:** Interobserver and intraobserver reliabilities of Doppler indices

Doppler indices	Intra-observer reliability			Inter-observer reliability
	Correlation coefficient			Correlation coefficient
	Operator1	Operator2	Operator3	
**MCA-PSV**	0.998	0.999	0.998	0.987
**DV-E**	0.990	0.951	0.859	0.821
**MV-E/A**	0.992	0.981	0.945	0.864
**TV-E/A**	0.956	0.996	0.964	0.965
**oICT**	0.981	0.944	0.977	0.924
**oIRT**	0.984	0.987	0.898	0.884
**oET**	0.994	0.980	0.987	0.948
**oLV-MPI**	0.975	0.952	0.939	0.830
**pICT**	0.974	0.981	0.980	0.952
**pIRT**	0.955	0.980	0.931	0.908
**pET**	0.953	0.978	0.983	0.961
**pLV-MPI**	0.924	0.944	0.908	0.875
**TI**	0.994	0.993	0.950	0.898
**PF**	0.986	0.985	0.921	0.851
**RV-MPI**	0.992	0.980	0.928	0.892

*CPR, cerebroplacental ratio; DV-E, early diastolic filling time of ductus venosus (sec); DV-PIV, pulsatility index for vein of ductus venosus; DV-S/a, systolic/atrial wave ratio of ductus venosus; MCA-PI, pulsatility index of middle cerebral artery; MCA-PSV, peak systolic velocity of middle cerebral artery (cm/sec); MV-E/A, E/A ratio of flow across mitral valve; oICT, isovolumetric contraction time measured by placing the caliper at the beginning of valve clicks (sec); pICT, isovolumetric contraction time measured by placing the caliper at the peak of valve clicks (sec); oIRT, isovolumetric relaxation time measured by placing the caliper at the beginning of valve clicks (sec); pIRT, isovolumetric relaxation time measured by placing the caliper at the peak of valve clicks (sec); oLV-MPI, myocardial performance index of left ventricle measured by placing the caliper at the beginning of valve clicks; pLV-MPI, myocardial performance index of left ventricle measured by placing the caliper at the peak of valve clicks; PF,; time from pulmonary valve opening to pulmonary valve closure (sec); RV-MPI, myocardial performance index of right ventricle; TI, time from tricuspid valve closure to tricuspid valve opening (sec); TV-E/A, E/A ratio of flow across tricuspid valve; UA-PI, pulsatility index of umbilical artery*

### The comparison and correlation of two LV-MPI techniques

**Fig. 5:**
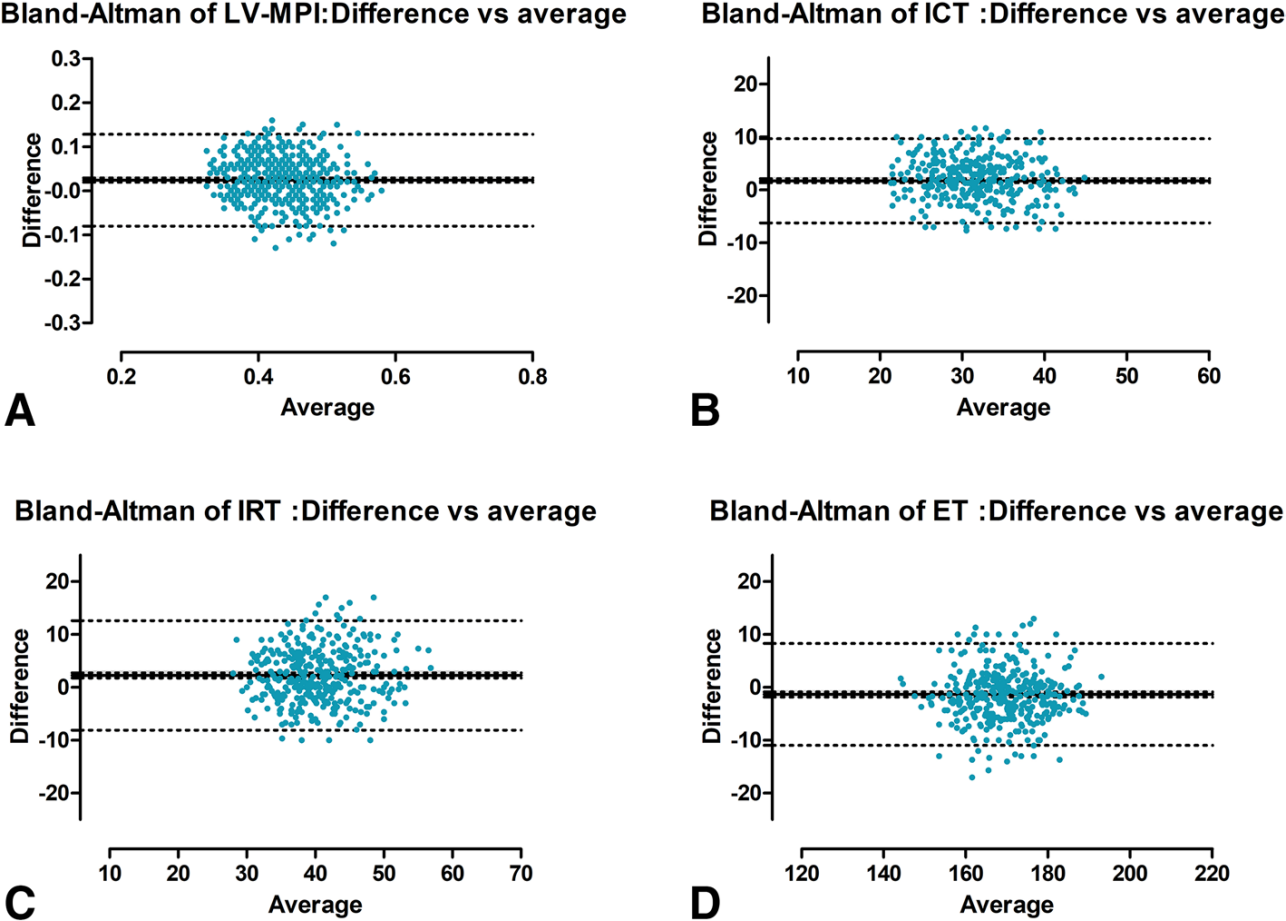
Bland–Altman graphs representing the agreement between the measurement at the beginning of valve clicks and the measurement at the peak of valve clicks of the left myocardial performance index (LV-MPI) (**A**), isovolumetric contraction time (ICT) (**B**), isovolumetric relaxation time (IRT) (**C**), and ejection time (ET) (**D**). The mean differences of the two methods of measurement (solid horizontal lines) were 0.024 (95% CI, 0.019 to 0.029) for LV-MPI, 1.74 (95% CI, 1.35 to 2.13) for ICT, 2.27 (95% CI, 1.77 to 2.77) for IRT, and − 1.34 (95% CI, − 1.81 to − 0.87) for ET with 95% limits of agreement (LOA, dashed horizontal lines) of − 0.080 to 0.128 for LV-MPI, − 6.21 to 9.69 for ICT, − 8.09 to 12.63 for IRT, and − 10.97 to 8.29 for ET

Although the values from the two caliper placement techniques were significantly correlated (r = 0.614, *P* < 0.001 for ICT; r = 0.575, *P* < 0.001 for IRT; r = 0.767, *P* < 0.001 for ET; r = 0.591, *P* < 0.001 for LV-MPI), the differences between oICT and pICT, oIRT and pIRT, and oLV-MPI and pLV-MPI were statistically significant (*P* < 0.001 for all). After the normal distribution of the differences was confirmed, the agreement between the two techniques was defined by calculating the averages of the differences and 95% LOA. The Bland-Altman graph in Fig. 5A shows significantly higher values of oLV-MPI than pLV-MPI, with an average of 0.024. The oICT and oIRT were significantly greater than pICT and pIRT, with averages of 1.74 and 2.27, respectively (Fig. 5B and C). Conversely, oET was significantly less than pET, with an average of − 1.34 (Fig. 5D).

### The relationship between MPI and FHR

In this study, the FHR decreased with advancing gestation (r = − 0.34, *P* < 0.001). After transforming to Z-scores adjusted for gestational age, oLV-MPI, pLV-MPI, and RV-MPI were not significantly correlated with FHR (oLV-MPI, *P* = 0.19; pLV-MPI, *P* = 0.13; RV-MPI*, P* = 0.076).

### The relationship between MPI and DV indices

After transforming to Z-scores adjusted for gestational age, DV-E was negatively correlated with oLV-MPI and pLV-MPI, corresponding to a negative correlation with pICT and pIRT and a positive correlation with oET and pET, even without correlation with oICT and oIRT. On the other hand, no correlation between DV-E and RV-MPI was observed. Neither DV-PIV nor DV-S/a correlated with oLV-MPI, pLV-MPI, and RV-MPI (Table 4).

**Table 4:** Correlation coefficients of the association between z-score of the myocardial performance indices and z-score of the ductus venosus indices (*n* = 442)

variable	oLV-MPI z-score	pLV-MPI z-score	RV-MPI z-score
**DV-PIV z-score**	−0.036	−0.054	−0.023
**DV-S/a z-score**	−0.029	− 0.022	− 0.076
**DV-E z-score**	− 0.155*	− 0.219 †	− 0.033

*DV-E, early diastolic filling time of ductus venosus; DV-PIV, pulsatility index for vein of ductus venosus; DV-S/a, systolic/atrial wave ratio of ductus venosus; oLV-MPI, myocardial performance index of left ventricle measured by placing the caliper at the beginning of valve clicks; pLV-MPI, myocardial performance index of left ventricle measured by placing the caliper at the peak of valve clicks; RV-MPI, myocardial performance index of right ventricle*

*P < 0.01

*†P < 0.001*

## Discussion

This study provides normative values of fetal cardiocirculatory indices in MCDA twin fetuses. Most indices were different from those of singleton fetuses. Two caliper placement techniques for the LV-MPI measurements contributed to distinct values. MPI was unaffected by FHR. When corrected for gestational age, DV-E was inversely related to LV-MPI.

The change in UA-PI corresponds to reduced placental resistance with gestational age. On account of the relatively low placental index, UA-PI was higher in this twin population than singleton fetuses from previous studies (Fig. S1) [19, 24–28]. Additionally, the assessment of umbilical blood flow at the fetal end in this study, pursuant to the ISUOG guidelines, gave rise to much greater UA-PI than all others [13, 14, 17]. Although the alteration in MCA-PI of twin fetuses that increased until 32 weeks of gestation resembled that of singleton fetuses, the median values appeared to be lower (Fig. S1) [13, 14, 19, 26, 27, 29, 30]. Higher UA-PI along with lower MCA-PI and CPR is in conformity with an adaptive response of MC placenta, provoking fetal growth to lag behind that of singleton pregnancy [19, 26, 29–32]. Hence, a cut-off CPR of < 1 should not be used to determine the abnormality for a MC twin fetus, especially at a gestation age of less than 28 weeks. At the gestational age of 24–35 weeks, the MCA-PSV values of singleton and twin fetuses were comparable (Fig. S1) [13–15, 29, 30, 33–35]. To this extent, the MCA-PSV reference of singletons can be applied for fetal surveillance in MC twin pregnancy during this period. However, the values before 24 weeks were higher than those of singletons. The diagnosis of fetal anemia based on singleton data before 24 weeks of gestation should be guarded. This corroborates with the study by Klaritsch but not by Casati and Mulcahy [13–15]. The disparity in the results could be attributed to the retrospective nature of Casati’s study and the large number of sonographers in the study by Mulcahy.

With advancing gestation, the decreased cardiac afterload together with the enhanced maturation of ventricular diastolic function results in increased DV-S and DV-a with the narrowing velocity gradient between these two phases, conforming to the declined DV-PIV and DV-S/a. There has only been one published study reporting the gestational age-specific DV-PIV values of twin fetuses, approximating the values of our twin cohort [14]. This is the first study of DV-S/a in normal twin fetuses; however, the comparison was only with singleton studies [36–40]. Both DV-PIV and DV-S/a indices in this study were quantitatively less than those in singleton studies (Fig. S2) [27, 36–42]. The naturally increased resistance in MC placentae would affect the peak velocity in the DV (DV-S) and, to a lesser degree, the flow velocity of DV during atrial contraction (DV-a) attenuated by normal fetal cardiac contractility [38, 43]. DV-E is another parameter derived from the DV waveforms, being a proxy for fetal cardiac compliance. The positive correlation between DV-E and gestational age has been postulated to be due to the improvement of ventricular diastolic function with advancing gestation [42, 44–46]. Only two singleton studies in literature provided normal ranges of DV-E [47, 48]. A plateau of DV-E at 28 weeks’ gestation was evident in this study, deviating from the pattern of increasing DV-E during gestation in the singleton data (Fig. S2). This diversity could be translated to the distinct placental resistance between singleton and twin fetuses, especially in late gestation. The greater afterload from placental resistance can lead to higher end-systolic intracardiac pressure or residual volume, thereby shortening the early diastolic time of forward flow in DV.

The E/A ratio increased with advancing gestation by virtue of reduced placental resistance and improved cardiac compliance. Instinctively, these two factors have a greater influence on early diastolic flow or E-wave than active flow or A-wave [44]. In this way, E-wave velocity progressively increases throughout gestation, whereas A-wave velocity gradually increases and remains at a plateau after 30 weeks of gestation [39, 42, 45]. From the results of this study, E/A ratios of both MV and TV in twin fetuses were expressed in the same way as in singletons with comparable values, assuring equivalent diastolic function (Fig. S3) [39, 45, 49].

In this twin population, LV-MPI and RV-MPI increased throughout gestation. As gestation advances, more time is consumed for each cardiac cycle to eject the increased fetal blood volume. Together with the decreasing FHR during gestation, these induce prolonged ICT and IRT. Notably, ET was stable irrespective of gestational age. The developmental change in cardiac maturation throughout gestation would have a greater effect on ventricular systolic function to maintain ET even with greater blood volume [50]. Consequently, MPI has remarkably increased in late gestation. As deduced from this study, the caliper placement technique influenced the LV-MPI values. The ICT and IRT measured at the beginning of the valve clicks were greater than those measured at the peak of the valve clicks. In contrast, the former technique generated a lower ET. The thickness of the valve clicks engenders this discrepancy. The closing valve clicks on the opposite side of the flow direction are typically wider than the opening valve clicks on the same side. Time interval demarcation at the beginning of the valve clicks would result in a higher value of LV-MPI. Moreover, the inconsistency of valve click thickness supports the variability of LV-MPI values based on the beginning of valve clicks among several studies (Fig. S3) [16, 51, 52]. Compared to the only singleton study reporting LV-MPI values based on the peak of valve clicks, the values derived from this technique during the period of 18–28 weeks were unchanged (Fig. S3) [16]. This bolsters the caliper placement at the peak of the valve clicks to measure LV-MPI. After 28 weeks of gestation, the deviation of LV-MPI in twin fetuses from singletons was consistent with the DV-E pattern, explained by the marked difference in placental resistance between twin and singleton pregnancies in late gestation. It is not surprising that the LV-MPI and RV-MPI values in the study by Van Mieghem were noticeably lower, possibly due to the exclusion of valve click thickness in ICT, IRT, and TI measurements (Fig. S3) [11].

Z-scores adjusted for gestational age were used for analysis as a consequence of the association between FHR and gestational age. FHR in the range of 120–160 beats/min had no impact on the MPI z-scores. This implies that as long as the FHR is in the normal range, the calculated MPI should be interpreted based on gestational age without adjustment for FHR.

Among the DV indices in this study, only DV-E significantly correlated with LV-MPI, consistent with the dependence of both LV-MPI and DV-E on cardiac compliance. Bensouda et al. reported that the increased IRT in the recipients of TTTS results in the decreased early diastolic filling time, corresponding to DV-E in this study [20]. The insignificant correlation between DV-E and RV-MPI may be associated with an inadequate sample size. The estimation of early diastolic time in the DV waveform is more advantageous in cases of monophasic atrioventricular flow in which the E-wave at the level of the atrioventricular valve cannot be determined [53]. From these evidences, DV-E, which is more accessible than MPI, would be a vascular index for the initial evaluation of diastolic function in MCDA twin pregnancy. Further studies are warranted to validate its clinical application. In keeping with the study by Sanapo, neither DV-PIV nor DV-S/a correlated with MPI [54]. Although the a-wave velocity is affected by the ventricular diastolic function, the atrial contraction itself is another factor that can introduce bias in this relationship [42, 55]. This may limit the use of DV-PIV and DV-S/a to assess diastolic cardiac performance.

Limitations of this study include its cross-sectional design and lack of echocardiographic confirmation of neonatal cardiac function and data regarding the association of perinatal outcomes. Despite the supernormal population, it is necessary to exclude any maternal and fetal abnormalities that contribute to these Doppler indices. Notwithstanding these limitations, this is the first study designed to establish the reference values of cardiocirculatory indices necessary for surveillance in MC twin fetuses using robust statistical methods. The reference values were obtained using standardized techniques with high reproducibility. Since the consequence of ethnic diversity is likely to be small, these data would be generalizable to other ethnic populations [56]. Precisely, the measurement technique and setting should be taken into account for the clinical application of reference ranges.

## Conclusion

The reference ranges of cardiocirculatory indices specific to MCDA twin pregnancy were established in meticulous detail with quantitatively different values from singletons. This accentuates the clinical use of normal MC twin–specific ranges. LV-MPI also relied on the caliper placement technique, which is more favorable for placing the caliper at the peak of the valve clicks. In the physiological range of FHR, it is not necessary to correct GA-adjusted MPI for FHR. By virtue of the correlation between MPI and DV-E time, fetal diastolic function may be indirectly assessed in the DV waveform.

## Supplementary Information

**Additional file 1: Supplementary Table S1**. The number of fetuses in each gestational period.

**Additional file 2: Supplementary Table S2**. Predicted vascular Doppler indices of centiles by gestational age.

**Additional file 3: Supplementary Table S3**. Predicted E/A ratio of mitral and tricuspid valves of centiles by gestational age.

**Additional file 4: Supplementary Table S4**. Predicted left myocardial performance indices of centiles by gestational age.

**Additional file 5: Supplementary Table S5**. Predicted right myocardial performance indices of centiles by gestational age.

**Additional file 6: Supplementary Figure S1**. Comparison of median values of arterial Doppler indices of this study population and those of other previous studies: (A) the pulsatility index of umbilical artery (UA-PI) [12, 13, 18, 25–27], (B) the pulsatility index of middle cerebral artery (MCA-PI) [12, 13, 18, 25, 26, 28, 29], (C) the cerebroplacental ratio (CPR) [12, 18, 25, 28, 29], (D) the peak systolic velocity of middle cerebral artery (MCA-PSV) [12–14, 28, 29, 31–33].

**Additional file 7: Supplementary Figure S2**. Comparison of median values of venous Doppler indices of this study population and those of other previous studies: (A) the pulsatility index for vein of ductus venosus (DV-PIV) [13, 26, 34–36, 38–40], (B) the systolic/atrial wave ratio of ductus venosus (DV-S/a) [34–38], (C) the early diastolic filling time of ductus venosus (DV-E) [45, 46].

**Additional file 8: Supplementary Figure S3**. Comparison of median values of cardiac Doppler indices of this study population and those of other previous studies. (A) the E/A ratio of flow across mitral valve (MV-E/A) [37, 43, 47], (B) the E/A ratio of flow across tricuspid valve (TV-E/A) [37, 43, 47], (C) the myocardial performance index of right ventricle (RV-MPI) [10], (D) the myocardial performance index of left ventricle measured by placing the caliper at the beginning of valve clicks (oLV-MPI) [10, 15, 49, 50], (E) the myocardial performance index of left ventricle measured by placing the caliper at the peak of valve clicks (pLV-MPI) [10, 15].

## Data Availability

The datasets used and/or analysed during the current study are available from the corresponding author on reasonable request.

## Abbreviations

AV: Aortic valve
CPR: Cerebroplacental ratio
DV: Ductus venosus
DV-E: E-wave duration of ductus venosus
DV-PI: Pulsatility index for veins of ductus venosus
DV-S/a: Systolic/atrial wave ratio of ductus venosus
ET: Ejection time
FHR: Fetal heart rate
ICC: Intraclass correlation coefficient
ICT: Isovolumetric contraction time
IQR: Interquartile range
IRT: Isovolumetric relaxation time
LV-E/A: E/A ratio of the left ventricle
LV-MPI: Left myocardial performance index
MCA: Middle cerebral artery
MCA-PI: Pulsatility index of middle cerebral artery
MCA-PSV: Peak systolic velocity of middle cerebral artery
MCDA: Monochorionic diamniotic
MPI: Myocardial performance index
MV: Mitral valve
oET: original ET
oICT: original ICT
oIRT: original IRT
pET: peak-to-peak ET
PF: Pulmonary flow
pICT: peak-to-peak ICT
pIRT: peak-to-peak IRT
PV: Pulmonary valve
RV-E/A: Ratios of the right ventricle
RV-MPI: Right myocardial performance index
sFGR: Selective fetal growth restriction
TAPS: Twin anemia polycythemia sequence
TI: Tricuspid interval
TRAPS: Twin reversed arterial perfusion sequence
TTTS: Twin-to-twin transfusion syndrome
TV: Tricuspid valve
UA: Umbilical artery
UA-PI: Pulsatility indices of umbilical artery
