# Supplementary material for: Reference values for fetal Doppler-based cardiocirculatory indices in monochorionic-diamniotic twin pregnancy
Source: BMC Pregnancy Childbirth. 2021 Nov 30;21:797. doi: 10.1186/s12884-021-04255-w (PMC8630902; doi:10.1186/s12884-021-04255-w)
Supplement: Supplementary file 1 — Additional file 1: Supplementary Table S1. The number of fetuses in each gestational period. [file 12884_2021_4255_MOESM1_ESM.docx]

| Gestational period (weeks) | number |
| --- | --- |
| 18 – 19 ^6/7^ | 50 |
| 20 – 21 ^6/7^ | 50 |
| 22 – 23 ^6/7^ | 50 |
| 24 – 25 ^6/7^ | 50 |
| 26 – 27 ^6/7^ | 50 |
| 28 – 29 ^6/7^ | 50 |
| 30 – 31 ^6/7^ | 46 |
| 32 – 33 ^6/7^ | 50 |
| 34 – 35 ^6/7^ | 46 |
