# Supplementary material for: Reference values for fetal Doppler-based cardiocirculatory indices in monochorionic-diamniotic twin pregnancy
Source: BMC Pregnancy Childbirth. 2021 Nov 30;21:797. doi: 10.1186/s12884-021-04255-w (PMC8630902; doi:10.1186/s12884-021-04255-w)
Supplement: Supplementary file 2 — Additional file 2: Supplementary Table S2. Predicted vascular Doppler indices of centiles by gestational age. [file 12884_2021_4255_MOESM2_ESM.docx]

| DV-E | p95 | 0.072 | 0.073 | 0.074 | 0.075 | 0.075 | 0.076 | 0.077 | 0.077 | 0.078 | 0.078 | 0.078 | 0.078 | 0.078 | 0.078 | 0.078 | 0.078 | 0.078 | 0.078 |
| --- | --- | --- | --- | --- | --- | --- | --- | --- | --- | --- | --- | --- | --- | --- | --- | --- | --- | --- | --- |
|  | p50 | 0.055 | 0.056 | 0.057 | 0.058 | 0.059 | 0.059 | 0.06 | 0.061 | 0.061 | 0.061 | 0.062 | 0.062 | 0.062 | 0.062 | 0.062 | 0.062 | 0.061 | 0.061 |
|  | p5 | 0.039 | 0.04 | 0.041 | 0.041 | 0.042 | 0.043 | 0.043 | 0.044 | 0.044 | 0.045 | 0.045 | 0.045 | 0.045 | 0.045 | 0.045 | 0.045 | 0.045 | 0.044 |
| DV-S/a | p95 | 2.831 | 2.767 | 2.708 | 2.653 | 2.601 | 2.554 | 2.51 | 2.469 | 2.432 | 2.398 | 2.366 | 2.338 | 2.313 | 2.29 | 2.27 | 2.252 | 2.238 | 2.225 |
|  | p50 | 2.107 | 2.06 | 2.015 | 1.974 | 1.936 | 1.901 | 1.868 | 1.838 | 1.81 | 1.784 | 1.761 | 1.74 | 1.721 | 1.704 | 1.689 | 1.676 | 1.665 | 1.656 |
|  | p5 | 1.568 | 1.533 | 1.5 | 1.469 | 1.441 | 1.415 | 1.39 | 1.368 | 1.347 | 1.328 | 1.311 | 1.295 | 1.281 | 1.268 | 1.257 | 1.248 | 1.239 | 1.233 |
| DV-PI | p95 | 1.024 | 0.989 | 0.958 | 0.929 | 0.902 | 0.878 | 0.856 | 0.835 | 0.817 | 0.801 | 0.786 | 0.773 | 0.761 | 0.751 | 0.743 | 0.736 | 0.73 | 0.725 |
|  | p50 | 0.661 | 0.639 | 0.618 | 0.599 | 0.582 | 0.567 | 0.552 | 0.539 | 0.527 | 0.517 | 0.507 | 0.499 | 0.491 | 0.485 | 0.479 | 0.475 | 0.471 | 0.468 |
|  | p5 | 0.427 | 0.412 | 0.399 | 0.387 | 0.376 | 0.366 | 0.356 | 0.348 | 0.34 | 0.334 | 0.327 | 0.322 | 0.317 | 0.313 | 0.309 | 0.306 | 0.304 | 0.302 |
| MCA-PSV | p95 | 35.81 | 36.29 | 36.9 | 37.63 | 38.5 | 39.5 | 40.62 | 41.88 | 43.26 | 44.78 | 46.42 | 48.19 | 50.1 | 52.13 | 54.29 | 56.58 | 59 | 61.55 |
|  | p50 | 24.87 | 25.35 | 25.96 | 26.69 | 27.56 | 28.56 | 29.68 | 30.94 | 32.32 | 33.84 | 35.48 | 37.25 | 39.16 | 41.19 | 43.35 | 45.64 | 48.06 | 50.61 |
|  | p5 | 13.93 | 14.41 | 15.02 | 15.75 | 16.62 | 17.62 | 18.74 | 20 | 21.38 | 22.9 | 24.54 | 26.31 | 28.22 | 30.25 | 32.41 | 34.7 | 37.12 | 39.67 |
| CPR | p95 | 1.237 | 1.317 | 1.397 | 1.477 | 1.556 | 1.634 | 1.71 | 1.784 | 1.854 | 1.921 | 1.983 | 2.04 | 2.092 | 2.138 | 2.178 | 2.21 | 2.236 | 2.254 |
|  | p50 | 0.886 | 0.943 | 1 | 1.057 | 1.114 | 1.17 | 1.224 | 1.277 | 1.327 | 1.375 | 1.42 | 1.461 | 1.498 | 1.531 | 1.559 | 1.583 | 1.601 | 1.614 |
|  | p5 | 0.634 | 0.675 | 0.716 | 0.757 | 0.798 | 0.838 | 0.877 | 0.914 | 0.95 | 0.985 | 1.017 | 1.046 | 1.073 | 1.096 | 1.116 | 1.133 | 1.146 | 1.156 |
| MCA-PI | p95 | 1.71 | 1.76 | 1.81 | 1.85 | 1.89 | 1.93 | 1.96 | 1.98 | 2.01 | 2.03 | 2.04 | 2.05 | 2.06 | 2.06 | 2.06 | 2.05 | 2.04 | 2.03 |
|  | p50 | 1.32 | 1.37 | 1.42 | 1.46 | 1.5 | 1.54 | 1.57 | 1.6 | 1.62 | 1.64 | 1.65 | 1.66 | 1.67 | 1.67 | 1.67 | 1.67 | 1.66 | 1.64 |
|  | p5 | 0.93 | 0.98 | 1.03 | 1.08 | 1.11 | 1.15 | 1.18 | 1.21 | 1.23 | 1.25 | 1.27 | 1.28 | 1.28 | 1.29 | 1.28 | 1.28 | 1.27 | 1.25 |
| UA-PI | p95 | 1.82 | 1.78 | 1.75 | 1.71 | 1.68 | 1.64 | 1.61 | 1.58 | 1.55 | 1.52 | 1.49 | 1.47 | 1.44 | 1.42 | 1.4 | 1.38 | 1.36 | 1.35 |
|  | p50 | 1.5 | 1.46 | 1.42 | 1.38 | 1.35 | 1.32 | 1.28 | 1.25 | 1.22 | 1.2 | 1.17 | 1.14 | 1.12 | 1.1 | 1.07 | 1.06 | 1.04 | 1.02 |
|  | p5 | 1.17 | 1.13 | 1.1 | 1.06 | 1.02 | 0.99 | 0.96 | 0.93 | 0.9 | 0.87 | 0.84 | 0.82 | 0.79 | 0.77 | 0.75 | 0.73 | 0.71 | 0.69 |
| GA | | 18 | 19 | 20 | 21 | 22 | 23 | 24 | 25 | 26 | 27 | 28 | 29 | 30 | 31 | 32 | 33 | 34 | 35 |
