# Supplementary material for: Reference values for fetal Doppler-based cardiocirculatory indices in monochorionic-diamniotic twin pregnancy
Source: BMC Pregnancy Childbirth. 2021 Nov 30;21:797. doi: 10.1186/s12884-021-04255-w (PMC8630902; doi:10.1186/s12884-021-04255-w)
Supplement: Supplementary file 3 — Additional file 3: Supplementary Table S3. Predicted E/A ratio of mitral and tricuspid valves of centiles by gestational age. [file 12884_2021_4255_MOESM3_ESM.docx]

| GA | MV-E/A | | | TV-E/A | | |
| --- | --- | --- | --- | --- | --- | --- |
|  | p5 | p50 | p95 | p5 | p50 | p95 |
| 18 | 0.47 | 0.59 | 0.71 | 0.51 | 0.62 | 0.73 |
| 19 | 0.48 | 0.6 | 0.72 | 0.52 | 0.63 | 0.74 |
| 20 | 0.49 | 0.61 | 0.73 | 0.54 | 0.65 | 0.75 |
| 21 | 0.5 | 0.62 | 0.74 | 0.55 | 0.66 | 0.77 |
| 22 | 0.51 | 0.63 | 0.75 | 0.56 | 0.67 | 0.78 |
| 23 | 0.53 | 0.65 | 0.77 | 0.57 | 0.68 | 0.79 |
| 24 | 0.54 | 0.66 | 0.78 | 0.58 | 0.69 | 0.8 |
| 25 | 0.55 | 0.67 | 0.79 | 0.59 | 0.7 | 0.81 |
| 26 | 0.56 | 0.68 | 0.8 | 0.6 | 0.71 | 0.82 |
| 27 | 0.57 | 0.69 | 0.81 | 0.61 | 0.72 | 0.83 |
| 28 | 0.58 | 0.7 | 0.82 | 0.62 | 0.73 | 0.84 |
| 29 | 0.59 | 0.71 | 0.83 | 0.64 | 0.75 | 0.85 |
| 30 | 0.6 | 0.72 | 0.84 | 0.65 | 0.76 | 0.87 |
| 31 | 0.61 | 0.73 | 0.85 | 0.66 | 0.77 | 0.88 |
| 32 | 0.62 | 0.74 | 0.86 | 0.67 | 0.78 | 0.89 |
| 33 | 0.64 | 0.76 | 0.88 | 0.68 | 0.79 | 0.9 |
| 34 | 0.65 | 0.77 | 0.89 | 0.69 | 0.8 | 0.91 |
| 35 | 0.66 | 0.78 | 0.9 | 0.7 | 0.81 | 0.92 |
