# Supplementary material for: Reference values for fetal Doppler-based cardiocirculatory indices in monochorionic-diamniotic twin pregnancy
Source: BMC Pregnancy Childbirth. 2021 Nov 30;21:797. doi: 10.1186/s12884-021-04255-w (PMC8630902; doi:10.1186/s12884-021-04255-w)
Supplement: Supplementary file 4 — Additional file 4: Supplementary Table S4. Predicted left myocardial performance indices of centiles by gestational age. [file 12884_2021_4255_MOESM4_ESM.docx]

| pLV-MPI | p95 | 0.487 | 0.489 | 0.491 | 0.494 | 0.498 | 0.501 | 0.505 | 0.51 | 0.515 | 0.52 | 0.526 | 0.532 | 0.539 | 0.547 | 0.554 | 0.563 | 0.572 | 0.581 |
| --- | --- | --- | --- | --- | --- | --- | --- | --- | --- | --- | --- | --- | --- | --- | --- | --- | --- | --- | --- |
|  | p50 | 0.389 | 0.391 | 0.393 | 0.395 | 0.398 | 0.401 | 0.404 | 0.407 | 0.411 | 0.416 | 0.42 | 0.426 | 0.431 | 0.437 | 0.443 | 0.45 | 0.457 | 0.465 |
|  | p5 | 0.311 | 0.312 | 0.314 | 0.316 | 0.318 | 0.32 | 0.323 | 0.326 | 0.329 | 0.332 | 0.336 | 0.34 | 0.344 | 0.349 | 0.354 | 0.36 | 0.365 | 0.371 |
| pIRT | p95 | 44.55 | 45.12 | 45.68 | 46.26 | 46.84 | 47.43 | 48.03 | 48.64 | 49.25 | 49.87 | 50.5 | 51.14 | 51.78 | 52.43 | 53.09 | 53.76 | 54.44 | 55.13 |
|  | p50 | 35.56 | 36 | 36.46 | 36.92 | 37.38 | 37.85 | 38.33 | 38.81 | 39.3 | 39.8 | 40.3 | 40.81 | 41.32 | 41.84 | 42.37 | 42.9 | 43.44 | 43.99 |
|  | p5 | 28.37 | 28.73 | 29.09 | 29.46 | 29.83 | 30.21 | 30.59 | 30.97 | 31.36 | 31.76 | 32.16 | 32.56 | 32.97 | 33.39 | 33.81 | 34.24 | 34.67 | 35.11 |
| pICT | p95 | 39.33 | 39.51 | 39.72 | 39.95 | 40.2 | 40.48 | 40.78 | 41.11 | 41.47 | 41.85 | 42.26 | 42.7 | 43.17 | 43.66 | 44.19 | 44.76 | 45.35 | 45.98 |
|  | p50 | 28.92 | 29.05 | 29.2 | 29.37 | 29.56 | 29.76 | 29.99 | 30.23 | 30.49 | 30.77 | 31.07 | 31.4 | 31.74 | 32.11 | 32.5 | 32.91 | 33.35 | 33.81 |
|  | p5 | 21.26 | 21.36 | 21.47 | 21.6 | 21.73 | 21.88 | 22.05 | 22.23 | 22.42 | 22.63 | 22.85 | 23.08 | 23.34 | 23.61 | 23.89 | 24.2 | 24.52 | 24.86 |
| oLV-MPI | p95 | 0.516 | 0.515 | 0.515 | 0.516 | 0.517 | 0.519 | 0.521 | 0.524 | 0.527 | 0.532 | 0.536 | 0.542 | 0.548 | 0.555 | 0.563 | 0.571 | 0.58 | 0.59 |
|  | p50 | 0.425 | 0.424 | 0.424 | 0.425 | 0.426 | 0.427 | 0.429 | 0.431 | 0.434 | 0.438 | 0.442 | 0.446 | 0.451 | 0.457 | 0.463 | 0.47 | 0.478 | 0.486 |
|  | p5 | 0.35 | 0.35 | 0.35 | 0.35 | 0.351 | 0.352 | 0.353 | 0.355 | 0.358 | 0.361 | 0.364 | 0.368 | 0.372 | 0.377 | 0.382 | 0.387 | 0.394 | 0.4 |
| oIRT | p95 | 47.74 | 48.28 | 48.83 | 49.38 | 49.94 | 50.51 | 51.08 | 51.66 | 52.24 | 52.84 | 53.43 | 54.04 | 54.65 | 55.27 | 55.9 | 56.53 | 57.17 | 57.82 |
|  | p50 | 37.94 | 38.37 | 38.8 | 39.24 | 39.69 | 40.14 | 40.59 | 41.05 | 41.52 | 41.99 | 42.47 | 42.95 | 43.43 | 43.93 | 44.42 | 44.93 | 45.44 | 45.95 |
|  | p5 | 30.15 | 30.49 | 30.84 | 31.19 | 31.54 | 31.9 | 32.26 | 32.63 | 33 | 33.37 | 33.75 | 34.13 | 34.52 | 34.91 | 35.3 | 35.7 | 36.11 | 36.52 |
| oICT | p95 | 41.4 | 41.17 | 41 | 40.87 | 40.79 | 40.77 | 40.79 | 40.86 | 40.98 | 41.15 | 41.37 | 41.64 | 41.96 | 42.32 | 42.74 | 43.21 | 43.72 | 44.29 |
|  | p50 | 32.33 | 32.11 | 31.93 | 31.81 | 31.73 | 31.7 | 31.72 | 31.8 | 31.92 | 32.09 | 32.31 | 32.57 | 32.89 | 33.26 | 33.68 | 34.14 | 34.66 | 35.22 |
|  | p5 | 23.27 | 23.04 | 22.87 | 22.74 | 22.67 | 22.64 | 22.66 | 22.73 | 22.85 | 23.02 | 23.24 | 23.51 | 23.83 | 24.2 | 24.61 | 25.08 | 25.59 | 26.16 |
| GA | | 18 | 19 | 20 | 21 | 22 | 23 | 24 | 25 | 26 | 27 | 28 | 29 | 30 | 31 | 32 | 33 | 34 | 35 |
