# Supplementary material for: Reference values for fetal Doppler-based cardiocirculatory indices in monochorionic-diamniotic twin pregnancy
Source: BMC Pregnancy Childbirth. 2021 Nov 30;21:797. doi: 10.1186/s12884-021-04255-w (PMC8630902; doi:10.1186/s12884-021-04255-w)
Supplement: Supplementary file 5 — Additional file 5: Supplementary Table S5. Predicted right myocardial performance indices of centiles by gestational age. [file 12884_2021_4255_MOESM5_ESM.docx]

| GA | TI | | | RV-MPI | | |
| --- | --- | --- | --- | --- | --- | --- |
|  | p5 | p50 | p95 | p5 | p50 | p95 |
| 18 | 213.04 | 235.81 | 258.57 | 0.251 | 0.373 | 0.495 |
| 19 | 215.6 | 238.37 | 261.14 | 0.26 | 0.382 | 0.504 |
| 20 | 217.97 | 240.74 | 263.51 | 0.268 | 0.39 | 0.512 |
| 21 | 220.15 | 242.92 | 265.68 | 0.276 | 0.398 | 0.52 |
| 22 | 222.13 | 244.9 | 267.66 | 0.284 | 0.406 | 0.528 |
| 23 | 223.92 | 246.68 | 269.45 | 0.291 | 0.413 | 0.535 |
| 24 | 225.51 | 248.28 | 271.04 | 0.297 | 0.419 | 0.541 |
| 25 | 226.91 | 249.68 | 272.44 | 0.303 | 0.425 | 0.547 |
| 26 | 228.11 | 250.88 | 273.65 | 0.309 | 0.431 | 0.553 |
| 27 | 229.12 | 251.89 | 274.66 | 0.314 | 0.436 | 0.558 |
| 28 | 229.94 | 252.7 | 275.47 | 0.318 | 0.44 | 0.562 |
| 29 | 230.56 | 253.32 | 276.09 | 0.322 | 0.444 | 0.566 |
| 30 | 230.98 | 253.75 | 276.52 | 0.325 | 0.447 | 0.569 |
| 31 | 231.22 | 253.98 | 276.75 | 0.328 | 0.45 | 0.572 |
| 32 | 231.25 | 254.02 | 276.79 | 0.33 | 0.452 | 0.574 |
| 33 | 231.1 | 253.86 | 276.63 | 0.332 | 0.454 | 0.576 |
| 34 | 230.74 | 253.51 | 276.28 | 0.333 | 0.455 | 0.577 |
| 35 | 230.2 | 252.97 | 275.73 | 0.334 | 0.456 | 0.578 |
