# Supplementary figures and images for: Reference values for fetal Doppler-based cardiocirculatory indices in monochorionic-diamniotic twin pregnancy
Source: BMC Pregnancy Childbirth. 2021 Nov 30;21:797. doi: 10.1186/s12884-021-04255-w (PMC8630902; doi:10.1186/s12884-021-04255-w)

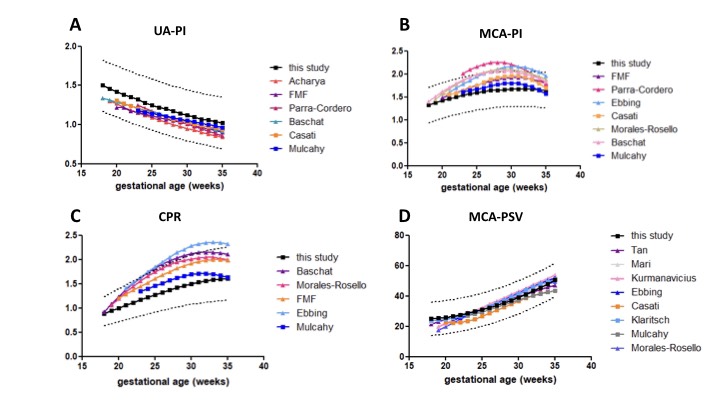

Supplement: Supplementary file 6 — Additional file 6: Supplementary Figure S1. Comparison of median values of arterial Doppler indices of this study population and those of other previous studies: (A) the pulsatility index of umbilical artery (UA-PI) [12, 13, 18, 25–27], (B) the pulsatility index of middle cerebral artery (MCA-PI) [12, 13, 18, 25, 26, 28, 29], (C) the cerebroplacental ratio (CPR) [12, 18, 25, 28, 29], (D) the peak systolic velocity of middle cerebral artery (MCA-PSV) [12–14, 28, 29, 31–33]. [file 12884_2021_4255_MOESM6_ESM.jpg]

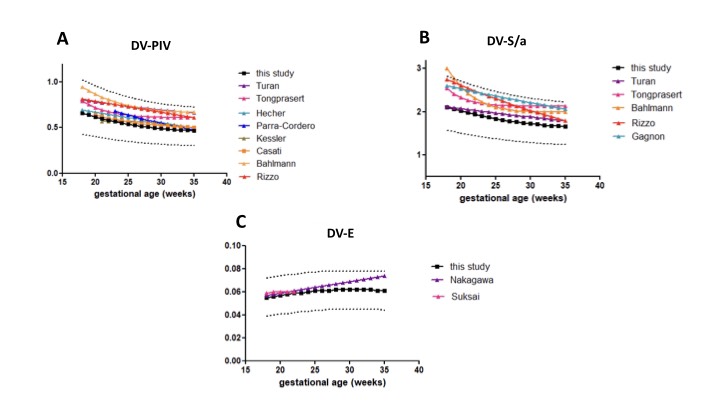

Supplement: Supplementary file 7 — Additional file 7: Supplementary Figure S2. Comparison of median values of venous Doppler indices of this study population and those of other previous studies: (A) the pulsatility index for vein of ductus venosus (DV-PIV) [13, 26, 34–36, 38–40], (B) the systolic/atrial wave ratio of ductus venosus (DV-S/a) [34–38], (C) the early diastolic filling time of ductus venosus (DV-E) [45, 46]. [file 12884_2021_4255_MOESM7_ESM.jpg]

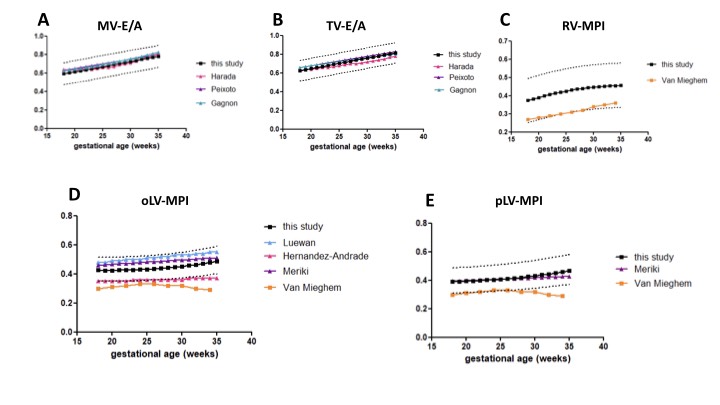

Supplement: Supplementary file 8 — Additional file 8: Supplementary Figure S3. Comparison of median values of cardiac Doppler indices of this study population and those of other previous studies. (A) the E/A ratio of flow across mitral valve (MV-E/A) [37, 43, 47], (B) the E/A ratio of flow across tricuspid valve (TV-E/A) [37, 43, 47], (C) the myocardial performance index of right ventricle (RV-MPI) [10], (D) the myocardial performance index of left ventricle measured by placing the caliper at the beginning of valve clicks (oLV-MPI) [10, 15, 49, 50], (E) the myocardial performance index of left ventricle measured by placing the caliper at the peak of valve clicks (pLV-MPI) [10, 15]. [file 12884_2021_4255_MOESM8_ESM.jpg]
